# Low-to-moderate alcohol intake and breast cancer risk in Chinese women

**DOI:** 10.1038/bjc.2011.302

**Published:** 2011-08-09

**Authors:** M Zhang, C D J Holman

**Affiliations:** 1 School of Population Health, The University of Western Australia, 35 Stirling Highway, Crawley, Perth, Western Australia 6009, Australia

**Keywords:** alcohol consumption, breast cancer, Chinese women, hormone receptor

## Abstract

**Background:**

Despite extensive investigation of the association between alcohol consumption and breast cancer risk, effect of low-to-moderate alcohol intake on breast cancer incidence has been inconsistent.

**Methods:**

A case–control study was conducted in China, 2004–2005 to examine the association by menopausal status, oestrogen (ER) and progesterone receptor (PR) status of the tumour. There were 1009 incident cases with histologically confirmed breast cancer and 1009 age-matched controls recruited. We assessed alcohol consumption by face-to-face interview using a validated questionnaire and obtained tumour ER and PR status from pathology reports.

**Results:**

Low-to-moderate alcohol consumption was inversely associated with breast cancer risk. Compared with nondrinkers, the adjusted odds ratios (ORs) for alcohol <5 g per day were 0.41 (95% confidence interval 0.27–0.62) and 0.62 (0.48–0.79) in postmenopausal and premenopausal women, respectively. The inverse association was consistent for alcohol <15 g per day across hormone receptor status groups with ORs of 0.36–0.56 in postmenopausal women and 0.57–0.64 in premenopausal women. An exception was that alcohol ⩾15 g per day appeared to increase the risk of breast cancers with discordant receptor status in postmenopausal women, that is, ER+/PR− or ER−/PR+ (4.27, 1.57–11.65).

**Conclusion:**

We found that low-to-moderate alcohol intake was not associated with increased risk of breast cancer in pre- or postmenopausal Chinese women. Future studies are required to understand differences in effect of alcohol on breast cancers by tumour hormone receptor status.

Despite extensive research on the association between alcohol consumption and breast cancer risk, a consensus is not apparent, especially on the effect of low-to-moderate alcohol intake (Brown *et al*, 2010). Since a meta-analysis provided evidence for a dose-response relation, admittedly with a weak ascending slope, alcohol has been treated as a risk factor for breast cancer even at low-to-moderate daily intake levels (Longnecker, 1994). Alcohol consumption has been reported to be associated with higher risk of breast cancer incidence (Smith-Warner *et al*, 1998; Bagnardi *et al*, 2001; Terry *et al*, 2006). However, subsequent studies based on more detailed data on consumption habits have not consistently supported the finding, particularly in premenopausal women (Garland *et al*, 1999; Kinney *et al*, 2000; Kropp *et al*, 2001; Baumgartner *et al*, 2002; Nagata *et al*, 2007; Bessaoud and Daurès, 2008; Brown *et al*, 2010; Kabat *et al*, 2010). Thus, the effect of low-to-moderate alcohol intake on breast cancer risk remains contentious.

Little is known about the relationship between alcohol intake and breast cancer risk in Chinese women, a population that consumes alcohol at very low levels (WHO, 2004). WHO reported that the lifetime prevalence of abstainers was 61.2% and that only 2.1% of Chinese women consumed three or more standard drinks on a typical drinking day (WHO, 2004). There have been a few studies on alcohol consumption and breast cancer risk in Chinese women. One study in premenopausal Vietnamese and Chinese women reported that those who consumed alcohol had an increased risk of breast cancer compared with women who did not (Nichols *et al*, 2005). However, a recent study suggested that low alcohol intake was not related to increased breast cancer risk in Asian Americans including Chinese women (Brown *et al*, 2010).

Considering that alcohol consumption is one of the few modifiable factors associated with breast cancer risk, investigations to further clarify this issue are warranted. We conducted a case–control study in southeast China specifically to examine associations between alcohol intake, primarily low-to-moderate levels and breast cancer risk in the population using a validated questionnaire. The analyses were stratified according to pre- and postmenopausal status and the study also explored whether differences exist in the effect of alcohol according to oestrogen (ER) and progesterone receptor (PR) status of the tumour.

## Participants and methods

### Study design and participants

A hospital-based case–control study of breast cancer risk was conducted in Hangzhou, the capital city of Zhejiang Province, during July 2004 and September 2005. All participants were Chinese women resident in Zhejiang Province and aged between 20 and 87 years. Cases were identified from medical records in four teaching hospitals of the School of Medicine, Zhejiang University. All the participating hospitals were public hospitals with 500–2000 beds and received patients from all over the province. A total of 1009 female patients, who newly diagnosed with invasive ductal carcinomas or *in situ* carcinoma of the breast, were recruited to the study. All diagnoses were histopathologically confirmed after surgery. The diagnoses occurred no more than 1 year before the interview and there was no previous diagnosis of cancer at any site. The patients were excluded if breast cancer was neither the primary nor final diagnosis. All relevant hospital and laboratory pathology reports were reviewed daily to ensure the completion of recruitment. The proportion of lost or non-responding patients among the cases was 1.2%. Among 1009 cases recruited, we obtained information on ER status in 756 cases (74.9%) and PR status in 755 cases (74.8%) from pathology reports filed in the patients’ medical records of immunohistochemistry assays undertaken at the time of diagnosis. Oestrogen receptor levels obtained from the pathology reports referred to the *α*-subtype of the receptor.

During the same period of data collection, 1009 outpatients who remained healthy were selected consecutively in the participating hospitals as controls to match each case's age within 5-year age groups, using a daily update of the list of cases after they had consulted their doctors. Each control was recruited as the first in the matched age group to attend the breast clinic for routine preventive care and who consented to participate. The proportion of selected controls who participated was 98.7%. Potential control women were excluded if they had a diagnosis of any benign or neoplastic breast disease, or another malignant disease at the time of recruitment. The project received ethics clearance from both the Human Research Ethics Committee of The University of Western Australia and the Chinese hospital authorities.

### Questionnaire and interview

Subjects were briefed regarding the general aims of the study to investigate lifestyle factors. An appointment for an interview was made after obtaining their consent at initial contact. A face-to-face interview was then conducted by trained staff using a structured questionnaire that usually took 40–50 min. The cases were interviewed in breast surgery wards and most of them (91.6%) within 3 months after diagnosis, while the controls were interviewed in the outpatient clinic of the same hospital. A validated and reliable questionnaire was used to collect the information on: (a) demographic and lifestyle characteristics, for example, residential area, education, weight and height, smoking, alcohol consumption, tea drinking, and physical activity; (b) habitual dietary intake assessed by a 100-item food frequency questionnaire (FFQ); and (c) factors relevant to hormonal status, including menstrual history and menopausal status, reproductive and lactation history, hormone replacement treatment, history of use of oral contraceptives, other factors relevant to hormonal status, benign proliferative breast disease and family history of breast cancer. After interview, anthropometric measurements were requested of all participants.

Alcohol consumption was assessed using the FFQ to measure three different types of alcoholic beverages, that is, beer, wine and liquor. The frequency of consumption was assigned to one of nine categories of never or hardly ever, once a month, 2–3 times a month, once a week, 2–3 times a week, 4–6 times a week, once a day, twice a day and ⩾3 times a day. Information was also sought on quantities consumed of alcohol in ml. Standard drinking vessels used by Zhejiang residents were displayed during the interview to increase the accuracy of measurement. Alcohol consumption was based on a usual drinking pattern and a ‘reference’ recall period was set as 1 year before diagnosis in cases or interview in controls. If there was any recent change in habits or quitting drinking, information was sought on the respondent's habits before the change and the year of quitting.

The questionnaire was adapted from that used in our previous studies on cancers (Zhang *et al*, 2002). The questionnaire was first pre-tested on 51 adult Chinese women who recently migrated to Perth, Australia, to assess the feasibility, face and content validity. The participants in the preliminary study reported their alcohol consumption when they were in China. The feedback from the participants indicated that they could estimate frequency and quantity of beer, wine and liquor consumed without difficulty. A test–retest study was then undertaken to assess the reliability of the questionnaire. Another sample of 41 Chinese women residing in Hangzhou was interviewed twice within 11.3 (s.d. 6.2) weeks (Zhang *et al*, 2005). No significant difference was found in alcohol consumption variables between the two interviews. Intraclass correlations ranged from 0.51 for wine intake to 0.87 for beer intake. The results, shown in Table 1, thus suggested that the questionnaire was a suitable and reproducible instrument to measure alcohol exposure for Chinese women.

### Statistical analysis

All data were checked for completeness at the end of each interview. The data were coded and analysed using the SPSS version 17.0 (SPSS Inc., New York, NY, USA). Data collected by different interviewers were compared and we confirmed that no consistent difference in recording key variables, such as alcohol consumption, tea drinking and physical activity, occurred within cases or controls. The frequency and quantity variables derived from the FFQ were converted into daily intake (in ml) of beer, wine and liquor. Amounts of ethanol intake were calculated by assuming 10 g of ethanol per 285 ml of beer, per 100 ml of wine and per 30 ml of liquor based on a method used in a previous study (Kropp *et al*, 2001). Food consumption was adjusted for the edible portions of foods, cooking methods, seasonal factors and market availability (Whitemore *et al*, 1990). Total energy intake was estimated using Chinese Food Composition Tables (Institute of Nutrition and Food Hygiene, 1999). Intakes of folate in *μ*g were calculated based on daily food consumption, using an updated version of the USDA nutrient database (USDA, 2007). The mean daily intakes of folate were tabulated separately for case and control groups. The quantitative variables of folate intake were divided into tertiles based on the corresponding empirical distribution in controls, with the low tertile being the reference category. Some category variables were defined as: a total of 20 packs of cigarettes or more in a lifetime for tobacco smoking; and BMI calculated from reported body height and weight using the formula for Quetelet's index (expressed in kg m^–2^). Physical activity was expressed in weekly metabolic equivalent task (MET) hours (Zhang *et al*, 2003). Metabolic equivalent task scores 6, 4.5 and 2.5 were assigned respectively for vigorous, moderate and walking activity based on a compendium of physical activities (Ainsworth *et al*, 2000). Patients were defined as postmenopausal if they indicated that they had ceased having periods for at least 1 year before the interview. Those patients who had had a surgical menopause at age over 50 years were also assigned to the postmenopausal group.

Distributions of demographic characteristics and potential risk factors of cases and controls were compared using the *t-*test for continuous variables and *χ*^2^-test for categorical variables. Odds ratios (ORs) and 95% confidence intervals (CIs) for each level of alcohol intake were estimated using unconditional logistic regression including the matching factors as covariates. Univariate analysis was undertaken to screen potential explanatory variables for subsequent multivariate analysis. In each analysis, effects of alcohol of different patterns and intake levels were assessed within current drinkers, excluding ex-drinkers (1.2% of participants), with abstainers who never drank alcohol as a reference group. We examined potential confounders of the alcohol effect through use of stratified analysis by menopausal status (premenopausal *vs* postmenopausal). Separate sub-analyses were conducted for each subtype of tumour hormone status (ER and PR separately and jointly). Each fitted regression equation was adjusted for age, education, BMI, oral contraceptive use, hormone replacement therapy, breast cancer in first-degree relatives, total energy intake, folate intake, tea drinking and menopausal status (only included the models for all women). These potential confounders were included in the models, because either they emerged as risk factors for breast cancer in previous studies (Kropp *et al*, 2001) or because we observed some evidence of potential confounding in our data set by comparisons of univariate and multivariate analyses (Zhang *et al*, 2007, 2009). The proportion of *in situ* carcinoma was only 2.2% of all breast cancer patients in the study, making it impracticable to study them separately. Therefore, the patients with *in situ* carcinomas and invasive breast cancer were combined to form a single case group in analyses.

## Results

Selected demographic characteristics and lifestyle factors in cases with breast cancer and controls are compared in Table 2. Fewer breast cancer cases (33.1%) were current drinkers compared with controls (46.6%). Among current drinkers, the majority of participants (77% cases, 87% controls) consumed <10 g daily, in contrast to 39 cases (12%) and 16 controls (3%) who consumed ⩾30 g daily. Cases had less education and tea drinking, and more use of oral contraceptives. More of cases had a higher BMI at 5-years ago, breast cancer in a first-degree relative and energy intake. There was no difference between cases and controls in age at interview, locality, age at first full-term pregnancy, number of children breastfed, menopausal status, hormone replacement therapy, smoking, mean dietary folate intake and physical activity in terms of weekly MET-hours.

Table 3 reports associations between breast cancer risk and alcohol consumption. Compared with abstainers, the adjusted ORs in current drinkers were 0.66 (95% CI 0.53–0.84) and 0.55 (0.38–0.78) for premenopausal and postmenopausal women. A significant inverse association was observed in premenopausal women who drank wine only (0.30, 0.17–0.52), and postmenopausal women who consumed beer only (0.34, 0.15–0.75). Compared with nondrinkers, those who consumed alcohol <5 g per day had a reduced risk of breast cancer. The adjusted ORs (95% CIs) were 0.41 (0.27–0.62) and 0.62 (0.48–0.79) in postmenopausal and premenopausal women, respectively. There was a marginal significant increased risk 2.28 (1.00–5.20) for daily alcohol intake ⩾30 g in premenopausal women.

Table 4 shows the combined effect of dietary folate intake and alcohol consumption on breast cancer risk. Folate intake was associated with a decreased risk of breast cancer in women who were abstainers or consumed alcohol >0–<15 g daily. Among the women who consumed alcohol ⩾15 g daily, a decreased breast cancer risk was only observed in those who consumed high not low or middle tertile of folate intake (OR=0.38, 95% CI 0.20–0.72) and not in the low or middle tertiles.

Table 5 shows associations between alcohol intake and breast cancer within hormone receptor subtypes. Compared with nondrinkers, the effects of alcohol intake of <15 g daily were protective in all subtypes defined by ER and PR status. For daily alcohol intake of ⩾15 g, an increased but nonsignificant association was found for all tumour receptor subtypes.

Table 6 reports adjusted ORs and 95% CIs for alcohol intake and breast cancer risk by joint ER and PR status. Relative to nondrinkers, an inverse effect was associated with alcohol intake <15 g per day for tumours with concordant but not discordant receptor status, that is, those with ER+/PR+ and ER−/PR−. The inverse association was more pronounced in postmenopausal women. Daily alcohol intake ⩾15 g was significantly associated with tumours in postmenopausal women with discordant receptor status, that is, ER+/PR− or ER−/PR+ (4.27, 1.57–11.65). There was no significant association with any joint hormone receptor subtype in premenopausal women.

## Discussion

Despite extensive investigation of the association between alcohol consumption and breast cancer risk, the conclusion remains controversial especially for low-to-moderate alcohol intake (Brown *et al*, 2010). Some studies reported that alcohol consumption was associated with a higher risk of incident breast cancer (Smith-Warner *et al*, 1998; Bagnardi *et al*, 2001; Terry *et al*, 2006), while others based on more detailed consumption measures have found no relationship with breast cancer, particularly in premenopausal women (Garland *et al*, 1999; Kinney *et al*, 2000; Kropp *et al*, 2001; Baumgartner *et al*, 2002; Nagata *et al*, 2007; Bessaoud and Daurès, 2008; Brown *et al*, 2010; Kabat *et al*, 2010). This study was specifically designed to examine associations between low-to-moderate intake of alcohol and the risk of breast cancer by menopause status in the women and ER and PR status in the breast tumours. We observed that women who consumed alcohol at a low frequency and quantity had a reduced risk of breast cancer regardless of menopausal status. Compared with nondrinkers, those who consumed alcohol at <5 g per day had a reduced breast cancer risk in pre- and postmenopausal women. For all tumour receptor subtypes combined, a small nonsignificant increased risk of higher alcohol intake (⩾15 g daily) was generally associated with breast cancer in this study. There was no clear association between types of alcohol consumed and breast cancer risk, although significantly reduced risks from wine and beer drinking were observed in pre- and postmenopausal women, respectively.

The findings from this study that low-to-moderate alcohol intake was, if anything, inversely associated with risk of breast cancer are supported by results from other observational studies. One study recently reported that breast cancer risk was not significantly associated with alcohol drinking (0.9, 0.7–1.1) in Asian Americans including Chinese women, a population consuming alcohol at a low level with a median intake of 0.48 g per day in cases and 0.40 g per day in controls (Brown *et al*, 2010). Another study conducted in France found that women who had an average consumption of <1.5 drinks per day had a lower risk (0.58, 0.34–0.97) when compared with nondrinkers (Bessaoud and Daurès, 2008). Another study reported that alcohol intake of <148 g per week was associated with a reduced risk in non-Hispanic Whites (0.49, 0.35–0.69), and that the protective effect was present in both pre- and postmenopausal women (Baumgartner *et al*, 2002). Furthermore, a reduced risk of breast cancer was found in premenopausal German women, whose average ethanol intake was ⩽11 g per day defined for a lower-level consumption of alcohol (Kropp *et al*, 2001). A systematic review of three cohort studies and eight case–control studies concluded that there were inconsistent results regarding alcohol drinking and breast cancer risk in the Japanese population (Nagata *et al*, 2007).

Chinese women have presently a very low level of alcohol consumption. WHO reported that 61.2% of the population were lifetime abstainers and only 2.1% of them consumed three or more standard drinks on a typical drinking day although the WHO has noted that alcohol consumption in Asia is rising rapidly (WHO, 2004). In this study, 65.4% cases and 52.4% controls self-reported as lifetime abstainers, while the majority of current drinkers consumed <10 g of alcohol per day, thus providing an opportunity to evaluate the association of low-to-moderate alcohol intake and breast cancer risk with reasonable precision. However, for the opposite reason, our study may be unsuitable to assess the effect of higher alcohol intake on breast cancer as few participants consumed at levels ⩾30 g daily. Few other studies have investigated the association of breast cancer with alcohol consumption in Chinese women. One such study was performed in premenopausal Vietnamese and Chinese women and reported that those who consumed alcohol had an increased risk of breast cancer (1.85, 1.32–2.61) compared with nondrinkers but no details on alcohol intake available (Nichols *et al*, 2005). However, another study suggested that low alcohol intake was unrelated to breast cancer risk (0.9, 0.7–1.1) in Asian Americans including Chinese women (Brown *et al*, 2010).

It is unclear whether the relationship between alcohol consumption and breast cancer risk differs across ER and PR tumours subtype (Suzuki *et al*, 2008). In the study, we classified hormone receptor status separately and jointly and found that low-to-moderate alcohol intake was consistently associated with a reduced risk of all tumour receptor subtypes except where tumours had discordant receptor status, that is, ER+/PR− or ER−/PR+. This risk reduction was evident in both pre- and postmenopausal women, but was more pronounced in postmenopausal women. There was no association between higher alcohol intake (daily ⩾15 g) and breast cancer risk with the exception of where discordant receptor status existed in postmenopausal women. There is no known biological basis for this result and we are reluctant to place much emphasis of it as an isolated finding in just one study.

Our findings regarding hormone receptor status are inconsistent to some extent with the results of other research. One study found that moderate alcohol consumption was associated positively with breast cancer in postmenopausal women with hormone receptor-positive tumours (Lew *et al*, 2009). A meta-analysis uncovered that an increase in alcohol consumption of 10 g per day was associated with statistically significant increased risks for all ER+, all ER−, ER+PR+ and ER+PR− but not ER−PR− tumours (Suzuki *et al*, 2008).

The biological mechanisms of the effects of alcohol on breast cancer aetiology have been widely discussed but remain to be characterised by detailed evidence. Investigators have suggested that alcohol consumption increases the risk of breast cancer in women by influencing oestrogen metabolism (Ginsburg, 1999). Alcohol appears to raise circulating oestrogen levels in premenopausal women and has a much more pronounced effect in postmenopausal women taking oestrogen replacements than in those not on replacement therapy (Ginsburg, 1999). The promotion of the growth of breast cancer may also be explained by an increased production by the liver of an insulin-like growth factor associated with moderate alcohol intake (Yu *et al*, 2002). Moreover, alcohol may exhibit mutagenic and oxidative effects in breast tissue, affect folate levels and lead to damaged DNA (Dumitrescu and Shields, 2005). The investigation found that high intake of folate protect against the increased risk of breast cancer associated with high alcohol consumption in Chinese women, which supports the findings reported by previous studies (Baglietto *et al*, 2005; Beasley *et al*, 2010). Although several possible mechanisms link chronic alcohol consumption to breast cancer, it is possible that the critical effect of alcohol on breast cancer depends on a threshold level of exposure. Therefore, lack of adverse effects of low alcohol consumption on cancer found in our study and some others is plausible.

It is necessary to emphasis our study's limitations and strengths of our study when explain the finding. A strong feature was that detailed data were obtained on alcohol consumption habits as well as diet, lifestyle and factors relevant to hormonal status. A validated and reliable instrument with high intraclass correlations in our test–retest study (Zhang *et al*, 2005) and designed specifically for Chinese women was used to collect this information. Another feature of the study was the adjustment for caloric intake and other potential confounders such as hormone-related factors, which could affect the tumour hormone receptor status of breast cancers. The total energy intake of participants, which was derived from the FFQ, was comparable to other data on Zhejiang residents (Ge *et al*, 1995). Nevertheless, the case–control design may have also introduced certain sources of bias. The breast cancer cases were identified from medical records and interviewed in breast surgery wards, while the controls were randomly selected from outpatient clinics in the same hospitals by matching on the age of cases. A majority of patients are self-referred in China. Under the recruitment procedure, the cases and controls arose from the same populations of women using the hospitals and, thus, if a control was later to develop breast cancer it was highly likely that she would have been treated in the same hospital.

Selection bias was also minimised by selection procedures for controls that were unrelated to convenience or clinician contact. However, there was potential for selection bias from the choice of hospital controls. The type of hospital controls selected in the study could have differed from women who would have been treated in the hospital had they developed breast cancer. In our previous cancer case–control study, we recruited community controls in addition to hospital controls to evaluate differences in estimates of exposure distributions between two control groups. There was no difference in alcohol consumption, although some differences were found in the intake of salted vegetables and animal fat (Zhang *et al*, 2002). Another limitation of the study was that 25.2% cases had no ER/PR information. We assessed the effect of alcohol consumption on breast cancer risk using the cases without ER/PR information and the results obtained were similar to those using cases with ER/PR information.

Our research interest in alcohol consumption and breast cancer was apparent to the respondents at the time of the interview as detailed data were sought on consumption habits. However, in assessing the likelihood of any information bias, which we consider to have been low, it was relevant that no mention occurred in the popular media of any association between alcohol consumption and breast cancer before and at the time of the research. Although alcohol consumption, like other personal habits, can be reported by the subjects with reasonable accuracy, misclassification of exposure may still occur. However, such random errors are likely to bias results towards the null and would have been unlikely to account for the strongly inverse associations reported here. Exposure of cases to risk factors may change because of disease status. However, over 90% cases were newly diagnosed and interviewed within 3 months. It appears unlikely, therefore, that disease status materially affected the interview responses in reporting alcohol habits using a ‘reference’ recall period.

In conclusion, this study found that low alcohol intake (<5 g daily) was inversely associated breast cancer in Chinese women. When considered according to hormone receptor status, the inverse association was consistently observed for all breast tumour receptor subtypes except those with a discordant receptor status, that is, ER+/PR− or ER−/PR+. The reduced risk was evident in both pre- and postmenopausal women, but was more pronounced in postmenopausal women. An increased risk from daily alcohol intake of ⩾15 g was found for tumours with a discordant receptor status in postmenopausal women. Given the number of women affected by breast cancer worldwide and the widespread consumption of alcohol, future studies are justified to fully understand the different effects of alcohol at low, moderate and high levels of intake on different breast tumour receptor subtypes according to hormone receptor status, including possible biological mechanisms.

## Figures and Tables

**Table 1:** Reliability assessment for alcohol intake in the test–retest study

**Alcohol intake (ml per day)**	**First interview Mean (s.d.)**	**Second interview Mean (s.d.)**	**Pearson correlation**	**Intraclass correlation**
Beer	0.15 (0.49)	0.23 (0.81)	0.98	0.87
Wine	0.06 (0.21)	0.11 (0.56)	0.76	0.51
Liquor	0.03 (0.11)	0.02 (0.08)	0.71	0.67

**Table 2:** Selected characteristics of subjects with and without breast cancer

	**Cases subjects (*n*=1009)**	**Controls subjects (*n*=1009)**	***P-*value** a
Age at interview (years)	48.4±10.3	48.4±10.3	0.96
Resident in rural area	471 (46.7)	475 (47.1)	0.86
			
*Education*			<0.01
No formal education	183 (18.1)	151 (15.0)	
Primary	290 (28.7)	289 (28.6)	
Secondary	395 (39.2)	376 (37.3)	
Tertiary	141 (14.0)	193 (19.1)	
			
*Body mass index* *(kg m^–2^, 5-years ago)*			<0.01
<25	794 (78.7)	841 (83.3)	
⩾25	215 (21.3)	168 (16.7)	
			
No. of delivery of full-term pregnancy	1.70±1.2	1. 71±1.2	0.94
No. of children breastfed	1.61±1.1	1.67±1.2	0.24
Oral contraceptive use (year)	3.1±5.1	2.3±3.6	0.02
Hormone replacement therapy (year)	1.7±3.4	1.4±1.6	0.67
Postmenopausal status	337 (33.4)	338 (33.5)	0.96
Breast cancer in first-degree relatives	35 (3.5)	17 (1.7)	0.01
			
*Alcohol consumption*			<0.001
Abstainers	660 (65.4)	529 (52.4)	
Ex-drinkers	15 (1.5)	10 (1.0)	
Current drinkers	334 (33.1)	470 (46.6)	
			
*Alcohol intake (ethanol, g per day)*			<0.001
>0–<5	225 (67.4)	366 (77.9)	
5–<10	28 (8.4)	42 (8.9)	
10–<20	12 (3.6)	13 (2.8)	
20–<30	45 (13.5)	43 (9.1)	
⩾30	39 (11.7)	16 (3.4)	
			
Tea drinking	451 (44.7)	661 (65.5)	<0.001
Tobacco smoking	20 (2.0)	14 (1.4)	0.30
Physical activity (weekly MET-hour)	58.3±71.2	60.1±59.1	0.54
Folate intake (*μ*g per day)	577.5±168.5	579.2±164.7	0.82
Energy intake (kcal per day)	2184.0±569.3	2084.3±506.3	<0.001

Abbreviation: MET=metabolic equivalent task.

a Two-sided, *t-*test for continuous variables and *χ*^2^ test for categorical variables.

Values expressed as mean±s.d. or number (percent).

**Table 3:** Association between alcohol consumption and breast cancer risk

	**No. cases/controls**	**OR (95% CI)** a	***P-*value**
*Alcohol consumption*			
All women			
Abstainers	660/529	1.00^b^	
Ex-drinkers	15/10	1.34 (0.56–3.22)	0.51
Current drinkers	334/470	0.63 (0.52–0.76)	<0.001
			
Premenopausal women			
Abstainers	416/332	1.00^b^	
Ex-drinkers	10/4	2.44 (0.71–8.39)	0.16
Current drinkers	246/335	0.66 (0.53–0.84)	0.001
			
Postmenopausal women			
Abstainers	244/197	1.00^b^	
Ex-drinkers	5/6	0.68 (0.17–2.67)	0.58
Current drinkers	88/135	0.55 (0.38–0.78)	0.001
			
*Type of alcohol consumed*			
All women			
Abstainers	660/529	1.00^b^	
Beer only	82/103	0.65 (0.47–0.90)	0.01
Wine only	37/91	0.40 (0.26–0.60)	<0.001
Liquor only	30/30	0.81 (0.47–1.39)	0.44
			
Premenopausal women			
Abstainers	416/332	1.00^b^	
Beer only	70/81	0.73 (0.51–1.06)	0.10
Wine only	19/60	0.30 (0.17–0.52)	<0.001
Liquor only	19/18	0.73 (0.37–1.47)	0.38
			
Postmenopausal women			
Abstainers	244/197	1.00^b^	
Beer only	12/22	0.34 (0.15–0.75)	<0.01
Wine only	18/31	0.61 (0.32–1.16)	0.13
Liquor only	11/12	0.88 (0.36–2.16)	0.77
*Ethanol intake (g per day)*			
All women			
None	660/529	1.00^b^	
>0–<5	225/366	0.56 (0.45–0.69)	<0.001
5–<10	28/42	0.58 (0.35–0.98)	0.04
10–<20	12/13	0.89 (0.39–2.03)	0.78
20–<30	45/43	0.86 (0.54–1.37)	0.53
⩾30	39/16	2.33 (1.26–4.31)	<0.01
			
Premenopausal women			
None	416/332	1.00^b^	
>0–<5	175/265	0.62 (0.48–0.79)	<0.001
5–<10	20/27	0.62 (0.33–1.17)	0.14
10–<20	8/9	0.85 (0.31–2.34)	0.75
20–<30	32/29	0.86 (0.49–1.50)	0.59
⩾30	21/9	2.28 (1.00–5.20)	0.05
			
Postmenopausal women			
None	244/197	1.00^b^	
>0–<5	50/101	0.41 (0.27–0.62)	<0.001
5–<10	8/15	0.49 (0.19–1.24)	0.13
10–<20	4/4	0.97 (0.23–4.06)	0.96
20–<30	13/14	0.97 (0.42–2.26)	0.95
⩾30	18/7	2.29 (0.89–5.87)	0.09

Abbreviations: CI=confidence interval; OR=odds ratio.

a Estimates from unconditional logistic regression models included terms for age at interview (continuous), education (none, primary, secondary, tertiary), BMI (5-years ago), oral contraceptive use (never, ever), hormone replacement therapy (never, ever), breast cancer in first-degree relatives (no, yes), total energy intake (continuous), folate intake (continuous), tea drinking (no, yes) and menopausal status (no, yes; only for all women).

b Reference category.

**Table 4:** Combined effect of dietary folate intake and alcohol consumption on breast cancer risk

**Ethanol intake (g per day)**	**Folate intake (*μ*g per day)**	**No. Cases/ controls**	**OR (95% CI)** a	***P-*value^b^**
Abstainers	Low	245/177	1.00^b^	
	Middle	229/166	0.70 (0.51–0.95)	
	High	186/186	0.30 (0.20–0.44)	
				
>0–<15	Low	70/136	0.43 (0.30–0.61)	<0.001
	Middle	99/154	0.35 (0.25–0.50)	
	High	87/122	0.23 (0.15–0.36)	
				
⩾15	Low	21/18	0.93 (0.47–1.84)	
	Middle	21/12	0.99 (0.46–2.16)	
	High	36/28	0.38 (0.20–0.72)	

Abbreviations: CI=confidence interval; OR=odds ratio.

a Estimates from unconditional logistic regression models included terms for age at interview (continuous), education (none, primary, secondary, tertiary), BMI (5-years ago), oral contraceptive use (never, ever), hormone replacement therapy (never, ever), breast cancer in first-degree relatives (no, yes), total energy intake (continuous), folate intake (continuous), tea drinking (no, yes) and menopausal status (no, yes; only for all women).

b Reference category.

**Table 5:** Association between alcohol intake (g per day) and breast cancer risk by hormone receptor status

		**ER+**		**ER−**		**PR+**		**PR−**	
	**No. controls**	**No. cases**	**OR (95% CI)** a	**No. cases**	**OR (95% CI)** a	**No. cases**	**OR (95% CI)** a	**No. cases**	**OR (95% CI)** a
All women									
None	529	277	1.00^b^	215	1.00^b^	257	1.00^b^	234	1.00^b^
>0–<15	412	118	0.62 (0.48–0.81)	74	0.52 (0.38–0.70)	107	0.58 (0.44–0.76)	84	0.56 (0.42–0.75)
⩾15	58	29	1.13 (0.69–1.86)	32	1.56 (0.95–2.53)	28	1.11 (0.67–1.84)	34	1.60 (0.99–2.59)
									
Premenopausal women									
None	332	184	1.00^b^	128	1.00^b^	178	1.00^b^	133	1.00^b^
>0–<15	297	89	0.64 (0.47–0.87)	54	0.57 (0.40–0.83)	86	0.63 (0.46–0.86)	56	0.59 (0.41–0.85)
⩾15	38	19	1.03 (0.56–1.92)	19	1.47 (0.78–2.76)	22	1.21 (0.67–2.18)	17	1.35 (0.70–2.61)
									
Postmenopausal women									
None	197	93	1.00^b^	87	1.00^b^	79	1.00^b^	101	1.00^b^
>0–<15	115	29	0.56 (0.34–0.94)	20	0.37 (0.21–0.68)	21	0.46 (0.26–0.81)	28	0.47 (0.28–0.79)
⩾15	20	10	1.49 (0.63–3.51)	13	1.80 (0.80–4.05)	6	1.03(0.37–2.87)	17	2.04 (0.97–4.31)

Abbreviations: CI=confidence interval; OR=odds ratio.

a Estimates from unconditional logistic regression models included terms for age at interview (continuous), education (none, primary, secondary, tertiary), BMI (5-years ago), oral contraceptive use (never, ever), hormone replacement therapy (never, ever), breast cancer in first-degree relatives (no, yes), total energy intake (continuous), folate intake (continuous), tea drinking (no, yes) and menopausal status (no, yes; only for all women).

b Reference category.

**Table 6:** Association between alcohol intake (g per day) and breast cancer risk by joint hormone receptor status

		**ER+/PR+**		**ER−/PR−**		**ER+/PR− or ER−/PR+**	
	**No. controls**	**No. cases**	**OR (95% CI)** a	**No. cases**	**OR (95% CI)** a	**No. cases**	**OR (95% CI)** a
All women							
None	529	235	1.00^b^	192	1.00^b^	64	1.00^b^
>0–<15	412	92	0.55 (0.41–0.73)	58	0.46 (0.33–0.64)	41	1.06 (0.68–1.64)
⩾15	58	20	0.89 (0.51–1.56)	25	1.42 (0.84–2.41)	16	2.82 (1.47–5.42)
							
Premenopausal women							
None	332	160	1.00^b^	109	1.00^b^	42	1.00^b^
>0–<15	297	72	0.58 (0.42–0.81)	39	0.49 (0.32–0.74)	31	1.08 (0.64–1.81)
⩾15	38	16	0.99 (0.51–1.89)	14	1.34 (0.67–2.69)	8	1.96 (0.80–4.79)
							
Postmenopausal women							
None	197	75	1.00^b^	83	1.00^b^	22	1.00^b^
>0–<15	115	20	0.45 (0.25–0.81)	19	0.36 (0.20–0.64)	10	0.98 (0.43–2.25)
⩾15	20	4	0.76 (0.23–2.51)	11	1.62 (0.69–3.81)	8	4.27 (1.57–11.65)

Abbreviations: CI=confidence interval; OR=odds ratio.

a Estimates from unconditional logistic regression models included terms for age at interview (continuous), residential area (urban, rural), education (none, primary, secondary, tertiary), BMI (5-years ago), age at menarche (continuous), oral contraceptive use (never, ever), hormone replacement therapy (never, ever), breast cancer in first-degree relatives (no, yes), total energy intake (continuous), folate intake (continuous), smoking (no, yes), tea drinking (no, yes), physical activity (weekly MET-hour, continuous) and menopausal status (no, yes; only for all women).

b Reference category.
